# Knowledge, Attitudes, and Practices Regarding Rabies in El Jadida Region, Morocco

**DOI:** 10.3390/vetsci7010029

**Published:** 2020-03-01

**Authors:** Khadija Bouaddi, Abdelali Bitar, Mohammed Bouslikhane, Abdesslam Ferssiwi, Aziz Fitani, Philip Paul Mshelbwala

**Affiliations:** 1 Provincial Veterinary Service of El Jadida, National Food Safety Office, ONSSA, El Jadida 24000, Morocco; 2 Nutritional Physiopathology and Toxicology Team, Department of Biology, Faculty of Sciences, Chouaib Doukkali University, El Jadida 24000, Morocco; abdelali.bitar@gmail.com (A.B.); ferssiwi.a@ucd.ac.ma (A.F.); 3 Department of Pathology and Veterinary Public Health, Institute of Agronomy and Veterinary Medicine Hassan II, BP 6202, Rabat-Instituts, Rabat 10000, Morocco; bouslikhanemed@yahoo.fr; 4 Private Veterinarian, El Jadida 24000, Morocco; azizfitani@hotmail.com; 5 Department of Veterinary Medicine, Faculty of Veterinary Medicine, University of Abuja, Abuja 900001, Nigeria; philbwala@yahoo.com

**Keywords:** rabies, knowledge, attitude, practice, El Jadida, Morocco

## Abstract

The aim of this study was to evaluate the knowledge, attitudes, and practices regarding rabies in the El Jadida region, Morocco. We conducted a cross-sectional survey using a structured questionnaire among randomly selected residents across 24 study sites. In total, 407 respondents took part in the survey. The majority (367, 92%) were male and had no formal education (270, 66%). Some (118, 29%) believed that rabies does not affect humans. Most respondents (320, 79%) were aware that vaccination could prevent rabies, but nevertheless did not vaccinate their dogs (264, 64.9%) and allowed their dogs to roam freely in search of food. Some (52.8%) would visit traditional healers for treatment in the event of a dog bite incident. Age and educational level were found to be significantly associated with knowledge, attitudes, and practices (*p* < 0.05). Although respondents demonstrated some level of knowledge about rabies, overall this study reveals critical gaps in their attitudes and practices. These shortcomings may be associated with a low level of education. Therefore, decision-makers need a new approach to control rabies, with a special focus on public awareness and health education, in order to sustain rabies control programs.

## 1. Introduction

Rabies causes a viral encephalitis that kills over 50,000 people every year [1].The majority of victims are in developing countries of Africa and Asia [2]. Most cases are due to domestic dog bites; as such, controlling the infection in the dog population is the most cost-effective way of controlling rabies [3]. In Africa, most cases of rabies are neither identified nor reported [4,5], consequently posing a huge challenge for rabies surveillance and control. Upgrading public knowledge coupled with improving knowledge, attitudes, and practice (KAP) surveys could support the prevention and control of rabies [6,7,8,9].

Rabies is widely distributed in Morocco, with an average of 391 animal and 22 human cases each year [10]. The close relationship between the human and canine population is a major risk factor for disease occurrence [11]. There is a large dog population (about 2,798,126) and low vaccination rate (<20%) in Morocco; coupled with unrestricted movement and reproduction, these factors may complicate rabies control efforts [12]. El Jadida is second in Morocco in terms ofthe incidence of human rabies cases [13]. Reports of rabies are very frequent in El Jadida, with 23 deaths each year between 2000 and 2017, and 15,625 dog bite cases.

There has been no proper assessment of KAP as it relates to rabies among the residents of El Jadida. Evaluation of KAP regarding rabies among high-risk groups could serve as an epidemiological tool and a basis for the development of rabies health promotion and intervention strategies in Morocco. This study aims to evaluate knowledge about rabies in the rural population, and the impact of people’s attitudes and practices on the persistence of rabies in the province of El Jadida, Morocco, in order to understand the disease, with the goal of achieving the World Health Organization (WHO) 2030 elimination plan.

## 2. Materials and Methods

### 2.1. Study Area

**Figure 1:**
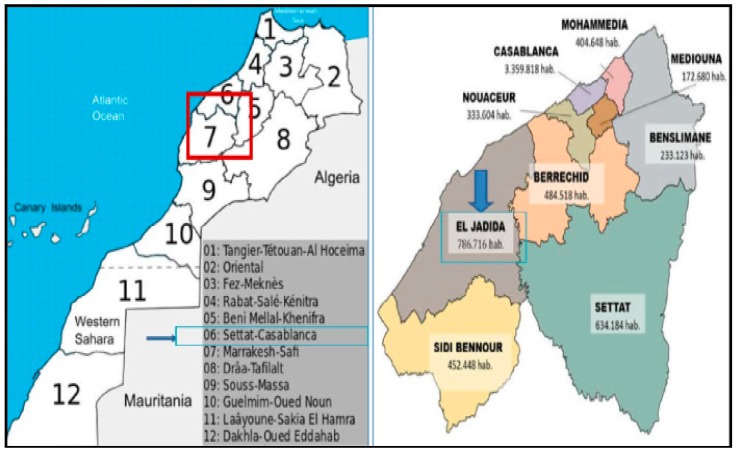
Map showing the geographical location of El Jadida province.

El Jadida province, one of 12 regions in Morocco, is part of the Casablanca–Settat region (Figure 1). It is among the most important agricultural provinces of the country. The proximity to the Atlantic has shaped the climate of the region, which is subject to maritime influences. In parallel with agriculture, most farmers practice intensive breeding of sheep, cattle, goats, and equines, in addition to the low court and beekeeping. Thanks to good weather conditions, livestock numbers in the region have increased slightly in recent years. Indeed, in 2012 the total livestock count, all species combined, was 3018 thousand head, representing 11% of the total count at the national level. It was distributed as follows: 2421 thousand head of sheep, 5497 thousand head of cattle, and 46 thousand head of goats [14].

### 2.2. Survey of the Rural Population

We conducted a cross-sectional study between March and May 2018 among residents of El Jadida. In total, 407 respondents in 24 rural communes took part in the survey.

### 2.3. Survey Method 

A structured questionnaire was designed and pretested using oral interviews among residents in the study area. After validation, we administered the questionnaire to randomly selected respondents within 48 douars (villages) from a formal list provided by the local authorities (communal government officers).

Before conducting the survey, three supervisors organized an orientation session for interviewers on the use of the questionnaire and the consent form. Each questionnaire consisted of 49 questions in four sections: biodemo graphic information, rabies knowledge, animal bite attitudes, and bite treatment practices. Only participants who gave their consent took part in the study. The Ethics Committee of the Nutritional Physiopathology and Toxicology Team, Department of Biology, Faculty of Sciences, Chouaib Doukkali University, approved the study.

### 2.4. Statistical Analysis

We used Epi Info (version 7.2.2.2 package) statistical software from the Centers for Disease Control and Prevention (CDC; Atlanta, GA, USA) to analyze the data. We present demographic variables using descriptive statistics and calculate the mean of KAP. We used chi-square test to test for association between KAP and certain sociodemo graphic factors (gender, age, education). We considered a *p*-value < 0.05 to be statistically significant.

## 3. Results

### 3.1. Community Members

Out of the 407 respondents, 376 (92.4%) were male, 290 (71.2%) were older than 40 years, 24 (5.9%) were civil servants, while 351 (86.2%) were farmers. Based on the level of education of respondents, 270 (66.3%) had no formal education, while only 19 (4.7%) had tertiary education. About 93.4%had at least one dog or more than one dog and 20.4% had a history of dog bite (Table 1). There was no significant difference in history of dog bite for those who currently owned a dog and those who did not (*p* > 0.05). Most people who had been bitten admitted that the bites were caused by neighbors’ dogs or stray dogs.

### 3.2. Knowledge about Rabies

Table 2 summarizes the survey results of the rural population’s knowledge of rabies. Indeed, all participants in this study had heard about rabies. Almost 75.9% of them confirmed the presence of rabies in Morocco and 89.2% knew that it is a fatal disease. Also, 93.4% knew that dogs are a common source of rabies virus in Morocco. More than 50% of respondents had little knowledge about rabies transmission and did not know that rabies affects the nervous system, while 30% believed that rabies does not affect humans. Only 45.9% were able to describe some rabies symptoms (severe behavioral changes and unexplained paralysis that worsens over time). In addition, 35% of respondents said that rabies does not affect other mammals.

### 3.3. Attitudes toward Dogs

According to the results in Table 3, 30% had the view that dogs can roam freely and 40% said they allowed their children to play with dogs irrespective of their vaccination status. About 54.3% admitted that dogs should be killed on sight if implicated in a biting incident. In addition, 64.9% of respondents reported that dogs should search freely for food and 52.1% had no objection to feeding unknown dogs.

### 3.4. Rabies Practices

About 44% of respondents did not know that post-exposure treatment starts with nonspecific treatment (washing the site of the bite with water and disinfectant). More than half would first seek treatment from traditional healers instead of going to a hospital. In response to the question about sterilizing dogs as a means of controlling the dog population, 45% of respondents did not agree with the practice and 54.3% preferred dog culling (Table 4).

### 3.5. Factors Associated with Rabies-Related KAP

**Figure 2:**
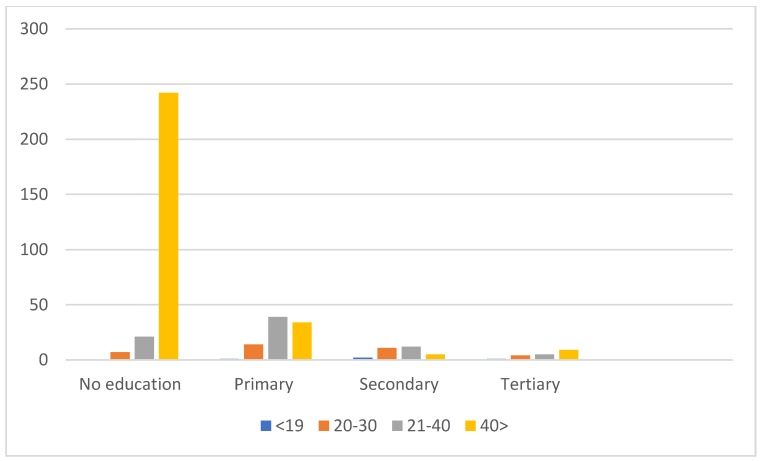
Distribution of respondents’ education level by age.

**Figure 3:**
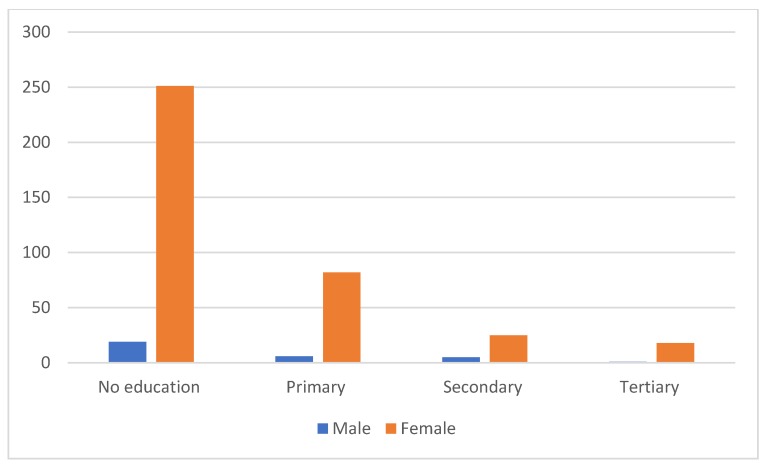
Distribution of respondents’ education level by gender.

Age and education were found to be significantly associated with KAP (*p* < 0.05) (Table 5). The level of KAP was low in adults compared to young people with a higher level of education (Figure 2). All respondents with a higher level of education had a good KAP with regard to rabies. Thus, level of education is closely associated with age (Figure 3).

## 4. Discussion

In this study, respondents demonstrated some level of knowledge about rabies signs; however, they were unable to describe the mode of rabies transmission. This is consistent with studies in other countries such as India [15], Malawi [16], Uganda [17], and Cameroon [18]. The low level of education as well as poverty, which is predominant in the study area, are possible reasons. Although the majority of respondents were aware that dogs are the major source of rabies in Morocco, they were oblivious to the role of other mammals. This finding is consistent with other reports in SriLanka [19] and Guatemala [20].

The level of awareness about rabies is influenced by several factors, such as age, sex, educational level [16], economic status [21], and religion [22]. In our study, no significant difference was found by gender, unlike a study done in North Carolina [23], which showed that women tended to have more knowledge. However, a study in Tanzania demonstrated that men were more knowledgeable when compared with women [24].

Our results indicate that young people had acceptable knowledge about rabies. This may be associated with the level of education among young people compared to adults. This finding is consistent with previous studies [15,23,25]. According to a survey in India, the proportion of respondents with adequate knowledge decreased with increasing age, less for women and those with less education. However, none of these associations was statistically significant in our study [26].

In this study, there was no association between dog possession and bite incidence. Most dog bite victim sad mitted that the bites were from neighbors’ dogs or stray dogs, unlike other studies [19,27,28], where dog owners were more likely to be bitten than non-owners. This is of great concern, as most of the dogs were reported to be unvaccinated and allowed to roam freely in the study area [10], which can provide a suitable condition for rabies transmission in El Jadida.

Certain respondents asserted that they had been bitten by dogs as children, which corroborates with evidence that children are the most vulnerable demographic for animal bites [5,29]. The increased frequency of dog bites among children may be due to their close relationship with dogs and their inability to protect themselves in case of dog attack. In addition, this study reveals that some families allow their children to play with dogs irrespective of their vaccination status. This may increase the incidence of bites and thus exposure to rabies.

Mass vaccination of dogs is necessary to interrupt the animal–human transmission cycle of rabies. According to WHO, vaccination coverage should reach at least 70% of the canine population in order to break this cycle [2]. In Morocco, the veterinary services organize free annual vaccination campaigns for dogs against rabies, but vaccination coverage rates have never exceeded 6% of the total canine population [8]. A possible explanation could be a low level of awareness among owners about the importance of dog vaccination. In addition, it might be difficult to restrain dogs that roam freely in search for food and present them at vaccination sites. In addition, people who are unaware of the risk of rabies may be less motivated to seek preventive care for their dogs.

Some respondents were completely unaware that they should wash a bite wound with soap and water for at least 15 min and seek medical help; this is a great public health concern. The percentage of respondents who were unaware is high when compared to other countries such as Cameroon, 18% [18]; Bangladesh, 2% [27]; and Ethiopia, 30.7% [30].

In Morocco, management of the canine population is carried out by eliminating stray dogs by either shooting them or poisoning them with strychnine. This is inefficient and counterproductive for vaccination programs [2], because dogs that are easy to vaccinate are also the easiest to kill, which leads to a reduction of the category of immunized dogs. Some respondents did not agree with dog sterilization and slaughtering as methods of preventing rabies in the dog population.

Although most respondents admitted awareness of the lethality of rabies disease, more than half confirmed that they consulted traditional healers in the event of a bite. In provinces where the incidence of rabies is high, such as El Jadida, a large majority of rural people strongly believe in the power of traditional healers, locally called *chorfa* or *maachat*. Spitting on the wound with the use of salt and isolating patients in dark rooms constitute their “therapy.” It is sad to note that one of the main recommendations by those healers to the families of exposed victims is that they should not consult a physician. Traditional practices differ between countries. For instance, in Bangladesh and India, traditional medicine includes the use of oils, salt, herbs, and red peppers in wounds [26,27]; in Nigeria, the use of herbs and concoctions [31]; and in Ethiopia, treatment includes herbs and a water locally called *tsebel* [30]. These traditional practices can cost people’s lives and are responsible for most cases of deadly human rabies [22,32]. Therefore, community awareness is crucial in the prevention and control of rabies.

Various factors are known to influence dogs’ roaming habits, including foraging, interacting with humans, and their sex and reproductive behavior. These factors may cause some dogs to wander more intensely, resulting in heterogeneous individual roaming patterns [3,33]. In our study, most of the respondents had no objection to letting their dogs roam freely to search for food, and many of the dogs were concentrated around poultry farms, as well as around areas where the local population throws the dead bodies of their sick animals.

Dogs that move freely are at high risk of contracting rabies virus because of the likelihood of contact with other feral dogs or mammals, subsequently contributing to the persistence of rabies [17,18]. Studies in Ethiopia and Tanzania found that a significant number of respondents stated that they would discard the carcasses of any of their animals suspected of rabies [22,24]. As a result, the presence of human-provided food resources increases the possibility of contact between dogs in the community and other stray dogs [34]. Responsible ownership is essential for population management of dogs, and rabies awareness campaigns should incorporate aspects of responsible dog ownership [35].

## 5. Conclusions

We identified an important knowledge gap and unsatisfactory attitudes and practices in El Jadida province regarding rabies. Increasing public knowledge of wound washing, seeking post-exposure prophylaxis, and the need to vaccinate dogs will likely improve rabies prevention and control in El Jadida, Morocco.

## Figures and Tables

**Table 1:** Sociodemo graphic characteristics of the population participating in the survey on knowledge, attitudes, and practices of rabies.

Variable		Frequency	Percentage
Age (years)	<19	4	1.0%
	20–30	36	8.8%
	31–40	77	18.9%
	>40	290	71.3%
Gender	Male	376	92.4%
	Female	31	7.6%
Educational level	No formal education	270	66.3%
	Primary	88	21.6%
	Secondary	30	7.4%
	Tertiary	19	4.7%
Marital status	Single	25	6.1%
	Married	369	90.7%
	Divorced	7	1.7%
	Widowed	6	1.5%
Occupation	Business man/woman	13	3.2%
	Civil servant	24	5.9%
	Unemployed	17	4.2%
	Farmer	351	86.2%
	Student	2	0.5%
Number of children	0	33	8.1%
	1	18	4.4%
	2	57	14.0%
	3	68	18.7%
	>3	231	56.8%
Number of dogs owned	0	27	6.6%
	1	42	10.3%
	2	176	43.2%
	3	61	15.0%
	>3	101	24.8%
Bitten by a dog before	No	324	79.6%
	Yes	83	20.4%

**Table 2:** Knowledge of rabies by study participants.

Statement	Agree		Disagree		Don’t Know	
	Frequency	Percentage	Frequency	Percentage	Frequency	Percentage
Rabiesexists in Morocco	309	75.9%	83	20.4%	15	3.7%
Pathogen that causes rabies affects the nerves	190	46.7%	11	2.7%	206	50.6%
Rabiescankill	363	89.2%	22	5.4%	22	5.4%
All animals can be infected with and transmit rabies virus	237	58.2%	142	34.9%	28	6.9%
Dogs are a possible common source of rabies virus in Morocco	380	93.4%	9	2.2%	18	4.4%
All humans can be infected with rabies	289	71.0%	94	23.1%	24	5.9%
Bites from an infected animal can spread rabies organism to another animal	303	74.5%	75	18.4%	29	7.1%
Rabies is spread through saliva of a rabid animal	243	59.7%	87	21.4%	77	18.9%
Age of first vaccination of dogs is 3 months	60	14.7%	89	21.9%	258	63.4%
An infected human can transmit rabies to another human through contact	218	53.6%	72	17.7%	117	28.8%
Dog bites increase your chances of getting rabies	293	72.0%	77	18.9%	37	9.1%
A friendly dog that suddenly turns aggressive may have rabies	346	85.0%	19	4.7%	42	10.3%
Excessive foamy salivation and tendency to bite anything are signs of rabies in dogs	187	46.0%	91	22.4%	129	31.7%
It is against the law to not vaccinate dogs against rabies	320	78.6%	15	3.7%	72	17.7%
Will present rabies vaccination certificate on request	291	76.6%	26	6.8%	63	16.6%
Dog registration and licensing can help in the control of rabies	302	74.2%	9	2.2%	96	23.6%
Vaccination of dogs against rabies should be repeated yearly	276	67.8%	15	3.7%	116	28.5%

**Table 3:** Attitudes of study participants.

Statement	Agree		Disagree		Don’t Know	
	Frequency	Percentage	Frequency	Percentage	Frequency	Percentage
I do not allow stray dogs to roam freely into my compound	282	69.3%	111	27.3%	14	3.4%
A dog that bites someone should be caught and killed	221	54.3%	96	23.6%	90	22.1%
It is not good to nurse an unknown sick dog	166	40.8%	212	52.1%	29	7.1%
If I am bitten by a dog, I will go to the hospital	288	70.8%	96	23.6%	23	5.7%
It is good to let dogs roam to get food because it makes them grow stronger	264	64.9%	102	25.1%	41	10.1%
It is inhumane/bad to confine your dog(s)	274	67.3%	84	20.6%	49	12.0%
It is good not play with unknown dogs	342	84.0%	38	9.3%	27	6.6%
Keeping dogs that are not vaccinated against rabies is dangerous and should be avoided	173	42.5%	105	25.8%	129	31.7%
Children should be allowed to play with dogs	163	40.1%	210	51.6%	34	8.4%

**Table 4:** Practices of study participants.

Question		Agree		Disagree		Don’t know	
		Frequency	Percentage	Frequency	Percentage	Frequency	Percentage
It is good to keep dogs?		262	64.4%	133	32.7%	12	3.0%
Is it good to vaccinate your dog(s)?		275	67.6%	12	3.0%	120	29.5%
It is good to wash dog bite wounds with soap?		228	56.0%	11	2.7%	168	41.3%
It is good to have a cage for your dog(s)?		144	35.4%	218	53.6%	45	11.1%
It is not a good practice to castrate/spay dogs?		183	45.0%	172	42.3%	52	12.8%
If a person is bitten by a dog, what should be done?	Do nothing	123	30.2%	206	50.6%	78	19.2%
	Take the victim to a chemist for treatment	5	1.2%	301	74.0%	101	24.8%
	Treat using traditional medicine	215	52.8%	181	44.5%	11	2.7%
	Take the victim to a veterinary clinic	240	58.97%	135	33.17%	32	7.86%
	Take the victim to a hospital	281	69.04%	103	25.31%	23	5.65%

**Table 5:** Association between knowledge, attitudes, and practices (KAP) and selected sociodemo graphic parameters among study participants.

Statement	Age (years)					Gender		Educational Level			
		<19	20–30	31–40	>40	Male	Female	No Formal Education	Primary	Secondary	Tertiary
All animals can be infected with and can transmit rabies virus	Agree	3 (75.0%)	27 (75.0%)	66 (85.7%)	141 (48.6%)	213 (56.6%)	24 (77.4%)	125 (46.3%)	73 (82.9%)	21 (70.0%)	18 (94.7%)
	Disagree	0 (0%)	3 (8.33%)	5 (6.5%)	134 (46.2%)	137 (36.4%)	5 (16.1%)	132 (48.9%)	5 (5.7%)	4 (13.3%)	1 (5.3%)
	Don’t know	1 (25.0%)	6 (16.7%)	6 (7.8%)	15 (5.2%)	26 (6.9%)	2 (6.4%)	13 (4.8%)	10 (11.4%)	5 (16.7%)	0 (0%)
	Chi-square	61.9				5.5		76.1			
	Df	6				2		6			
	*p*-value	0.0000				0.0634		0.0000			
Dogs are a possible common source of rabies virus in Morocco	Agree	3 (75.0%)	31 (86.1%)	65 (84.4%)	281 (96.9%)	352 (93.6%)	28 (90.3%)	259 (95.9%)	77 (87.5%)	25 (83.3%)	19 (100%)
	Disagree	1 (25.0%)	2 (5.6%)	4 (5.2%)	2 (0.7%)	7 (1.9%)	2 (6.4%)	2 (0.7%)	4 (4.5%)	3 (10.0%)	0 (0%)
	Don’t know	0 (0%)	3 (8.3%)	8 (10.4%)	7 (2.4%)	17 (4.5%)	1 (3.2%)	9 (3.3%)	7 (7.9%)	2 (6.7%)	0 (0%)
	Chi-square	29.0				2.9		18.8			
	Df	6				2		6			
	*p*-value	0.0001				0.24		0.0046			
Rabies is spread through saliva of a rabid animal	Agree	4 (100%)	17 (47.2%)	53 (68.8%)	169 (58.3%)	224 (59.6%)	19 (61.3%)	150 (55.6%)	55 (62.5%)	20 (66.7%)	18 (94.7%)
	Disagree	0 (0%)	4 (11.1%)	7 (9.1%)	76 (26.2%)	8 (21.3%)	7 (22.6%)	79 (29.3%)	5 (5.7%)	3 (10.0%)	0 (0%)
	Don’t know	0 (0%)	15 (41.7%)	17 (22.1%)	45 (15.5%)	72 (19.1%)	5 (16.1%)	41 (15.2%)	28 (31.8%)	7 (23.3%)	1 (5.3%)
	Chi-square	27.21				0.17		40.82			
	Df	6				2		6			
	*p*-value	0.0001				0.91		0.0000			
I do not allow stray dogs to roam freely into my compound	Agree	3 (75.0%)	31 (86.1%)	67 (87.0%)	181 (62.4%)	262 (69.7%)	20 (64.5%)	154 (57.0%)	82 (93.2%)	27 (90.0%)	19 (100%)
	Disagree	1 (25.0%)	3 (8.3%)	7 (9.1%)	100 (34.5%)	103 (27.4%)	8 (25.8%)	106 (39.3%)	3 (3.4%)	2 (6.7%)	0 (0%)
	Don’t know	0 (0%)	2 (5.6%)	3 (3.9%)	9 (3.1%)	11 (2.9%)	3 (9.7%)	10 (3.7%)	3 (3.4%)	1 (3.3%)	0 (0%)
	Chi-square	27.31				3.93		60.70			
	Df	6				2		6			
	*p*-value	0.0001				0.14		0.0000			
A dog that bites someone should be caught and killed	Agree	2 (50.0%)	21 (58.3%)	62 (80.5%)	136 (46.9%)	205 (54.5%)	16 (51.6%)	111 (41.1%)	73 (82.9%)	18 (60.0%)	19 (100%)
	Disagree	2 (50.0%)	7 (19.4%)	10 (13.0%)	77 (26.5%)	86 (22.9%)	10 (32.3%)	82 (30.4%)	8 (9.1%)	6 (20.0%)	0 (0%)
	Don’t know	0 (0%)	8 (22.22%)	5 (6.49%)	77 (26.55%)	85 (22.61%)	5 (16.13%)	77 (28.52%)	7 (7.95%)	6 (20.0%)	0 (0%)
	Chi-square	30.95				1.66		64.44			
	Df	6				2		6			
	*p*-value	0.0000				0.44		0.0000			
Keeping dogs that are not vaccinated against rabies is dangerous and should be avoided	Agree	3 (75.0%)	21 (58.3%)	45 (58.4%)	104 (35.9%)	161 (42.8%)	12 (38.7%)	80 (29.6%)	54 (61.4%)	22 (73.3%)	17 (89.5%)
	Disagree	1 (25.0%)	8 (22.2%)	10 (13.0%)	86 (29.7%)	94 (25.0%)	11 (35.5%)	90 (33.3%)	11 (12.5%)	3 (10.0%)	1 (5.26%)
	Don’t know	0 (0%)	7 (19.4%)	22 (28.6%)	100 (34.9%)	121 (32.2%)	8 (25.8%)	100 (37.0%)	23 (26.1%)	5 (16.7)	1 (5.3%)
	Chi-square	21.4				1.7		62.1			
	Df	6				2		6			
	*p*-value	0.0016				0.43		0.0000			
It is good to keep a dog	Agree	3 (75.0%)	23 (63.9%)	59 (76.6%)	177 (61.0%)	249 (66.2%)	13 (41.9%)	152 (56.3%)	72 (81.8%)	25 (83.3%)	13 (68.4%)
	Disagree	0 (0%)	9 (25.0%)	15 (19.5%)	109 (37.6%)	119 (31.6%)	14 (45.2%)	111 (41.1%)	13 (14.8%)	3 (10.0%)	6 (31.6%)
	Don’t know	1 (25.0%)	4 (11.1%)	3 (3.9%)	4 (1.4%)	8 (2.1%)	4 (12.9%)	7 (2.6%)	3 (3.4%)	2 (6.7%)	12 (2.9%)
	Chi-square	28.0				15.5		30.0			
	Df	6				2		6			
	*p*-value	0.0001				0.0004		0.0000			
It is good to wash dog bite wounds with soap	Agree	2 (50.0%)	18 (50.0%)	43 (55.8%)	165 (56.9%)	217 (57.7%)	11 (35.9%)	152 (56.3%)	44 (50.0%)	18 (60.0%)	14 (73.7%)
	Disagree	1 (25.0%)	1 (2.8%)	5 (6.5%)	4 (1.4%)	8 (2.1%)	3 (9.7%)	3 (1.1%)	8 (9.1%)	0 (0%)	0 (0%)
	Don’t know	1 (25.0%)	17 (47.2%)	29 (37.7%)	121 (41.7%)	151 (40.2%)	17 (54.8%)	115 (42.6%)	36 (40.9%)	12 (40.0%)	5 (26.3%)
	Chi-square	14.4				10.1		20.0			
	Df	6				2		6			
	*p*-value	0.0250				0.0065		0.0028			
If a person is bitten by a dog, treat using traditional medicine	Agree	1 (25.0%)	11 (30.6%)	23 (29.9%)	180 (62.1%)	194 (51.6%)	21 (67.7%)	188 (69.6%)	20 (22.7%)	6 (20.0%)	1 (5.3%)
	Disagree	3 (75.0%)	22 (61.1%)	51 (66.2%)	105 (36.2%)	171 (45.5%)	10 (32.3%)	76 (28.1%)	64 (72.7%)	23 (76.7%)	18 (94.7%)
	Don’t know	0 (0%)	3 (8.3%)	3 (3.9%)	5 (1.7%)	11 (2.9%)	0 (0%)	6 (2.2%)	4 (4.5%)	1 (3.3%)	0 (0%)
	Chi-square	37.8				3.4		95.4			
	Df	6				2		6			
	*p*-value	0.0000				0.18		0.0000			
